# mTOR Activation Is Required for the Proliferation of Reactive Astrocytes in the Hippocampus During Traumatic Brain Injury

**DOI:** 10.3390/biom16040555

**Published:** 2026-04-09

**Authors:** Lilesh Kumar Pradhan, Xiaoting Wang, Fang Yuan, Xiang Gao

**Affiliations:** Spinal Cord and Brain Injury Research Group, Department of Neurological Surgery, Stark Neurosciences Research Institute, Indiana University School of Medicine, 320 W. 15th Street, Indianapolis, IN 46202, USA; lkpradha@iu.edu (L.K.P.); xiaotingwang0122@gmail.com (X.W.); fyuan@iu.edu (F.Y.)

**Keywords:** traumatic brain injury, reactive astrocytes, astrocyte proliferation, hippocampus, mTORC1

## Abstract

Astrocytes undergo pronounced reactivity during traumatic brain injury (TBI); however, the temporal dynamics of this response and the signaling mechanisms regulating astrocyte proliferation remain incompletely defined. In this study, we characterized the spatiotemporal profile of astrocyte reactivity and proliferation in the hippocampus during TBI and investigated the involvement of mammalian target of rapamycin complex 1 (mTORC1) signaling in these processes. Using a mouse model of TBI, we found that injury triggered a rapid astrocytic response in the hippocampus, characterized by increased glial fibrillary acidic protein (GFAP) expression and morphological hypertrophy as early as 4 h post-injury. Astrocyte proliferation emerged subsequently, peaked during the acute phase (48 and 72 h), and declined to baseline levels at 7 days post-trauma, indicating a transient proliferative response during TBI. Concurrently, mTORC1 signaling was robustly activated in reactive astrocytes in the hippocampus and was specifically associated with proliferative reactive astrocytes during injury. Pharmacological inhibition of mTORC1 signaling with rapamycin significantly reduced reactive astrocyte proliferation during TBI without altering astrocytic hypertrophy. Together, these findings demonstrate that TBI induces a rapid but transient astrocyte activation and proliferation response in the hippocampus and that mTORC1 activation is required for the proliferation, but not the hypertrophic activation, of reactive astrocytes during traumatic brain injury.

## 1. Introduction

Traumatic brain injury (TBI) is a significant global health issue, leading to approximately 3.0 million emergency department visits, hospitalizations, and deaths in the United States alone in 2013 [1]. The socioeconomic burden is immense, costing affected families and society nearly $76.5 billion annually [2]. TBI survivors often experience a range of neurological and psychiatric complications, including cognitive impairments, epilepsy, depression, and an increased risk of neurodegenerative diseases such as Alzheimer’s disease, Parkinson’s disease, and dementia [3]. The hippocampus, a brain region crucial for memory and emotional regulation, is particularly vulnerable to TBI-related damage, contributing to persistent cognitive and emotional dysfunctions [4]. Network disconnection within the hippocampus, resulting from neuronal death, axonal injury, and synaptic degeneration, has been identified as a key factor underlying these deficits [5]. Despite extensive research efforts, no FDA-approved treatments are available, largely due to the lack of understanding of mechanisms underlying post-traumatic neurodegeneration and repair.

Astrocytes, which constitute 20–40% of total brain cells, play essential roles in neuronal function by maintaining metabolic support, regulating synaptic plasticity, releasing neurotrophic factors, and preserving blood–brain barrier (BBB) integrity [6,7,8]. Following brain injury, astrocytes undergo reactive astrogliosis, characterized by altered gene expression, hypertrophy, and proliferation [9]. This phenomenon has been widely observed in various neurological disorders, including Alzheimer’s disease, Huntington’s disease, and spinal cord injury [8]. Reactive astrocytes perform both beneficial and detrimental roles, including glial scar formation, BBB repair, inflammation modulation, and synaptic regulation [9]. However, the precise molecular mechanisms governing astrocyte reactivation remain poorly understood, hindering the ability to selectively manipulate their functions in vivo.

Unlike in the injured cortex, where reactive astrocytes primarily contribute to glial scar formation, astrocytes in the hippocampus exhibit a distinct response to TBI. Previous reports demonstrate that astrocyte proliferation significantly increases within the hippocampus during injury, despite the absence of a glial scar [10,11]. The mechanistic target of rapamycin (mTOR) signaling, particularly mTOR complex 1 (mTORC1), is a critical intracellular pathway regulating cell growth, proliferation, and survival [12]. Studies, including our previous work, have shown that mTORC1 signaling is significantly activated in the hippocampus during TBI [13,14]. Our findings suggest that mTORC1 activation is temporally and spatially correlated with astrocyte proliferation, highlighting its potential role in post-traumatic astrogliosis.

In this study, we aimed to elucidate the mechanisms underlying reactive astrocyte proliferation in the hippocampus during TBI. Also, we investigated whether mTORC1 activation is required for the proliferation of reactive astrocytes in the hippocampus during TBI, and we compared hippocampal and cortical responses to controlled cortical impact.

## 2. Materials and Methods

### 2.1. Animal Care

Male C57BL/6 mice (The Jackson Laboratory, Bar Harbor, ME, USA) were housed in a 12 h/12 h light–dark cycle environment. In this study, we used male mice to minimize potential variability associated with hormonal fluctuations during the estrous cycle, which can influence neuroinflammatory responses and cellular proliferation during brain injury. This approach is commonly used in initial mechanistic studies of TBI to reduce biological variability [15,16]. In addition, traumatic brain injury has a higher incidence in males compared to females in clinical populations, which further supports the relevance of studying male subjects in this context [17]. Access to food and water was provided *ad libitum*. A total number of 91 animals were used in this study. The sample size was calculated based on our previous study [13]. Mice aged 8–10 weeks with baseline body weight 22–28 g were included; animals were excluded if outside this range, if they showed pre-injury abnormalities or if they lost more than 15% body weight post-injury. The animals were randomly allocated to different groups, and the animals were evenly distributed across cages. The experimenters were blinded to the treatments, and the data analyst was blinded to experimental groups. Group identities were only revealed after completion of statistical analyses. The euthanasia method, which is consistent with the recommendations of the American Veterinary Medical Association (AVMA) Guidelines on Euthanasia, was used for the humane endpoint. All animal procedures were conducted in compliance with protocols approved by Indiana University Institutional Animal Care and Use Committees (IACUC; protocol #24039; approval period 5 June 2024–5 May 2027).

### 2.2. Controlled Cortical Impact (CCI)

Male mice at the age of 8–10 weeks were subjected to moderate CCI or sham surgeries as we previously described [18]. Briefly, mice were anesthetized with 2.5% avertin and fixed in a stereotactic frame (Kopf Instruments, Tujunga, CA, USA). A 4 mm craniotomy was performed at the center between the bregma and lambda, midway between the midline and temporalis muscle. The skullcap was carefully removed without disturbing the underlying dura. The electromagnetic impact piston was positioned perpendicular to the exposed cortical surface. For moderate TBI, the contact velocity was set at 3.5 m/s with 0.1 s duration at 1.0 mm deformation depth. After bleeding stopped, the wound was sutured. All surgeries were performed under strict temperature control, with a heating pad to maintain body temperature at 36–37 °C. Sham animals received a craniotomy without impact. The mice with moderate brain injury by CCI showed minor pain and distress after waking up from surgery. Slow-release Buprenorphine (3.25 mg/kg, Patterson Veterinary, Loveland, CO, USA) was administrated subcutaneously right after surgery for analgesia to minimize pain.

### 2.3. Drug Administration

Mice received 5-bromo-2′-deoxyuridine (BrdU, 100 mg/kg in saline, i.p., Sigma, St. Louis, MO, USA) injections at 0 h, 20 h, 44 h, 68 h, and 1 week post-TBI for BrdU incorporation studies. All the mice were perfused 4 h after BrdU injection, and the brains were collected to assess proliferation of activated astrocyte (*n* = 3 for each group). Additionally, rapamycin (2 mg/kg, Sigma, St. Louis, MO, USA) in a solution of 5% PEG400/4% ethanol and 5% Tween 80, i.p. was administered at 24, 48, and 68 h post-injury to inhibit mTORC1 signaling.

### 2.4. Tissue Processing

Mice were euthanized by transcardial perfusion with cold saline, followed by 4% paraformaldehyde (PFA) in PBS. Brains were removed, post-fixed overnight in PFA, and cryoprotected in 30% sucrose for 48 h. Serial 30 μm coronal sections were cut using a Leica CM 1950 cryostat (Leica Biosystems, Nussloch, Germany) and stored at −20 °C until they were processed for immunohistochemistry.

### 2.5. Immunohistochemistry

Brain sections were processed for immunostaining using a free-floating protocol. Briefly, sections were washed three times in phosphate-buffered saline (PBS) and incubated in blocking buffer containing 5% normal goat serum, 1% bovine serum albumin, and 0.1% Triton X-100 in PBS for 1 h at 4 °C. Sections were then incubated with primary antibodies diluted in blocking buffer overnight at 4 °C. Following three washings in PBS, sections were incubated in secondary antibodies at R.T. for 2 h. Then, sections were briefly stained with 4′,6-diamidino-2-phenylindole (DAPI, Sigma, St. Louis, Missouri, USA), washed three times in PBS, put on the slides, and mounted with Fluoromount-G (Southern Biotech, Birmingham, AL, USA). For bromodeoxyuridine (BrdU) incorporation analysis, sections were pretreated with 2 N HCl at room temperature for 1 h, neutralized in 0.1 M borate buffer (pH 8.5) for 20 min, and washed three times in PBS prior to blocking and immunostaining as described above. The primary antibodies used were anti-BrdU (rat, 1:200; AbD Serotec, Oxford, United Kingdom), anti-GFAP (mouse, 1:800; Sigma, St. Louis, MO, USA), and anti-phospho-S6 (rabbit, 1:200; Cell Signaling Technology, Danvers, MA, USA). Appropriate species-specific secondary antibodies conjugated to fluorophores (Jackson ImmunoResearch Laboratories, West Grove, PA, USA) were applied at a dilution of 1:400.

### 2.6. Cell Quantification

Cell counts were performed on the hippocampus, including CA1, CA3, and the dentate gyrus (DG) and the cortex, using a Zeiss Axiovert 200M inverted microscope (Oberkochen, Germany). Z-stack images were captured for each region of interest, and the number of BrdU-positive, GFAP-positive, and co-labeled cells (double) were counted using Adobe Photoshop 7.0. Briefly, images were acquired as 20× tiled mosaics with z-stacks, and maximum intensity projections were generated for analysis. Cell counts were then performed across these reconstructed images, encompassing the region of the hippocampus (including CA1, CA3, and the dentate gyrus). Specifically, three coronal brain sections (30 µm thick) centered around the epicenter of the injury (TBI) or corresponding anatomical region in sham (craniotomy) animals were analyzed. Quantifications were expressed as the average number of cells per section (*n* = 3 for each group).

### 2.7. Western Blot

Western blot was performed to examine pS6 expression in the hippocampus as previously described [13]. Briefly, 10-week-old male mice of two different groups, including wild type sham and TBI 72 h, were euthanized. The animals were transcardially perfused with cold saline (0.9% sodium chloride in sterile water), and brains were then removed immediately. The hippocampi were dissected out and transferred to centrifuge tubes containing 500 μL ice-cold T-PER™ Tissue Protein Extraction Reagent (Thermo Scientific, Waltham, MA, USA) and protease inhibitor cocktail (Roche, Basel, Switzerland) for homogenizing. Homogenized tissues were centrifuged at 12,000 rpm for 30 min at 4 °C. Protein concentrations of different samples were determined in the cleared lysates by Bradford assay (Bio-Rad, Hercules, CA, USA). To measure pS6 expression, the protein concentration of each sample was normalized to 1 mg/mL, and an aliquot containing 30 μg protein of each sample was run on SDS PAGE (10% [*w*/*v*] acrylamide) gel with a Tris-Glycine running buffer system. After electrophoresis, the proteins were transferred to nitrocellulose membranes using an electrotransferring unit (Bio-Rad) at 20 V overnight. Next day, the membranes were incubated in blocking solution (EveryBlot Blocking Buffer; 12010020, Bio-Rad) for 1 h at room temperature and probed with anti-pS6 antibody (rabbit, 1:1000; CST 4858L, Danvers, MA, USA) overnight at 4 °C. Meanwhile, an antibody against β-tubulin (mouse, 1:1000; Santa Cruz sc-80011, Cambridge, MA, USA) was probed for internal control. On the third day, a donkey anti-rabbit secondary antibody conjugated to an infrared dye (IRDye680, 1:5000; Li-Cor Bioscience, Lincoln, NE, USA) and a donkey anti-mouse secondary antibody conjugated to an infrared dye (IRDye800CW, 1:5000; Li-Cor Bioscience, Lincoln, NE, USA) were then applied for 1 h at room temperature. After drying, the membranes were imaged using the Li-Cor Odyssey Fc Imaging System (Lincoln, NE, USA), and pS6 and β-tubulin corresponding bands were quantified using ImageJ (v1.54g, Bethesda, MD, USA). After imaging, membranes were stripped using stripping buffer (Restore™ Western Blot Stripping Buffer, Thermo Scientific 21059, Waltham, MA, USA) for 30 min at room temperature, then re-blocked and re-probed with anti-S6 (rabbit, 1:1000; CST 2217S, Danvers, MA, USA) and β-tubulin (mouse, 1:1000; Santa Cruz sc-80011, Cambridge, MA, USA) overnight at 4 °C. The following day, appropriate IRDye-conjugated secondary antibodies (1:5000; Li-Cor) were applied for 1 h at room temperature. Membranes were imaged, and S6 and β-tubulin bands were quantified using ImageJ.

### 2.8. Statistics

Data were expressed as mean ± standard deviation. Statistical analyses were performed using one-way or two-way ANOVA followed by LSD post hoc test for multiple comparisons, or Student’s *t*-test for comparisons between two groups. A *p*-value of <0.05 was considered statistically significant.

## 3. Results

### 3.1. TBI Triggers Rapid Astrocyte Reactivity Followed by Transient Proliferation in the Hippocampus

Ten-week-old male C57BL/6 mice were subjected to TBI using a CCI model, with sham-operated mice serving as controls, as previously described [18]. Mice were sacrificed three days later to examine astrocyte responses in the hippocampus, a region particularly vulnerable to TBI-related cell loss [19] and closely linked to deficits in learning and memory [20]. Astrocyte activation was evaluated by immunostaining for GFAP.

**Figure 1:**
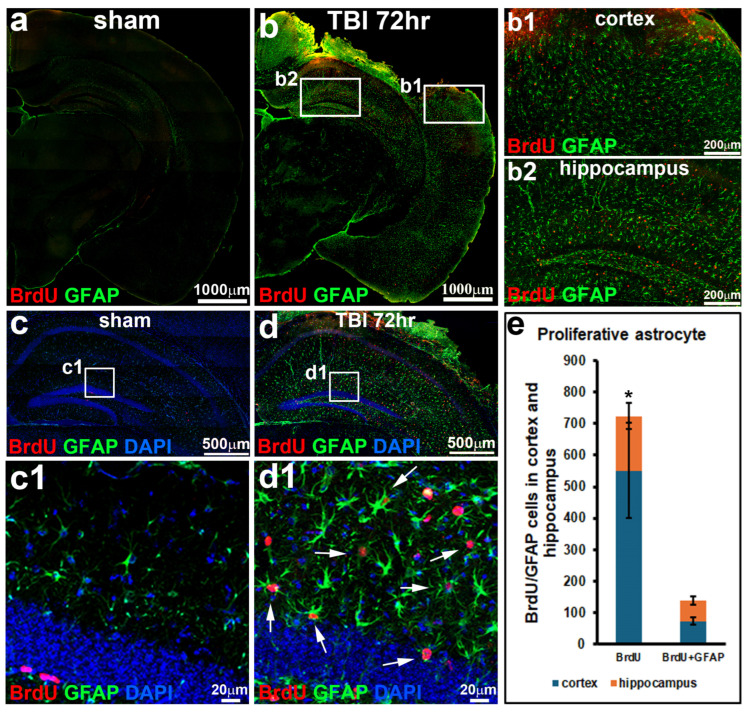
Proliferation of reactive astrocytes in hippocampus during TBI. (**a**,**b**) Immunostaining of GFAP (green) and BrdU (red) showing increase in reactivation and proliferation of astrocyte in cortex (**b1**) and hippocampus (**b2**) during TBI when compared to sham. (**c**,**d**) Proliferation of reactive astrocytes (GFAP-green and BrdU-red double positive cells, white arrows) in hippocampus. (**c1**,**d1**) Enlarge images of (**c**,**d**). (**e**) Quantification of BrdU and GFAP-BrdU double-positive cells in the cortex and hippocampus (*n* = 5 for each group, * *p* < 0.05).

In sham-operated mice, astrocytes in the hippocampus showed a typical resting appearance, with low levels of GFAP expression and thin, delicate processes (Figure 1a,c,c1). These cells were sparsely distributed throughout the hippocampus. In contrast, TBI led to a striking increase in the number of GFAP-positive astrocytes in the ipsilateral hippocampus (Figure 1b). During injury, astrocytes displayed classic features of activation, including enlarged cell bodies, thickened processes, and strong GFAP staining (Figure 1d,d1), consistent with astrocytic hypertrophy [21].

**Figure 2:**
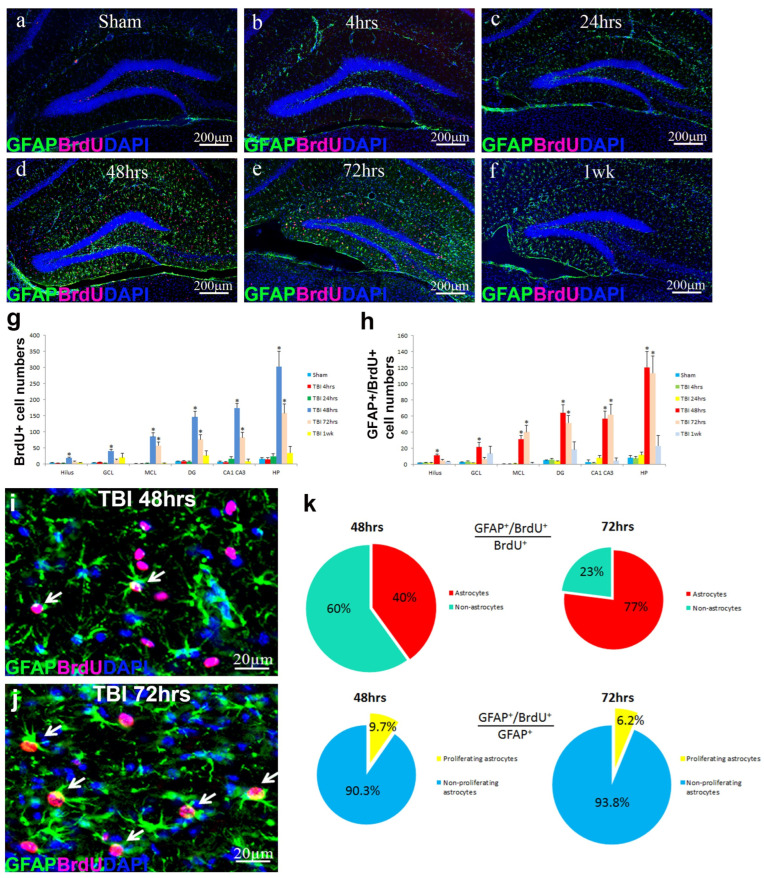
The pattern of astrocyte activation in the hippocampus during traumatic brain injury. (**a**–**f**) The alteration of GFAP/BrdU expression (protein) at different time points during TBI. Immunostaining with antibody against GFAP (green) and BrdU (red) demonstrated the time course of astrocyte reactivation and proliferation during TBI. (**g**) The number of BrdU-positive cells in the hippocampus and its sub-regions: CA1 + CA3 and dentate gyrus (DG) and its sub-regions, molecular layer (ML), granule cell layer (GCL), and hilus. (**h**) The number of GFAP-positive/BrdU-positive cells in hippocampus and its sub-regions: CA1 + CA3 and DG and its sub-regions, molecular layer (ML), granule cell layer (GCL), and hilus, reflecting the reactive astrocyte proliferation in these areas. (**i**,**j**) Co-localization of GFAP- and BrdU-positive cells peaking at 48 and 72 h post-TBI (white arrows). (**k**) Quantification of proliferating cells and reactive astrocytes in the hippocampus (*n* = 5 for each group, * *p* < 0.05).

In sham mice, BrdU-positive cells were rare and largely restricted to the subgranular zone, consistent with normal levels of neural progenitor proliferation [21,22], and most astrocytes did not incorporate BrdU (Figure 1a,c,c1). In contrast, TBI triggered a marked rise in BrdU-positive cells throughout the hippocampus (Figure 1b,d,d1). Importantly, at 72 h post-injury, 77% of these dividing cells also expressed GFAP (Figure 2j,k), indicating that reactive astrocytes not only undergo morphological changes during injury but also actively re-enter the cell cycle and proliferate. Interestingly, beside the injured site of the cortex (Figure 1b,b1), remarkable proliferating reactive astrocytes (GFAP-green and BrdU-red double-positive cells) in the hippocampus were also detected (Figure 1b,b2,d,d1). Quantitative analysis revealed that at 72 h post-TBI, nearly half of all proliferating reactive astrocytes were localized within the hippocampus (47.37 ± 1.51%, Figure 1e), highlighting the hippocampus as a major site of astrocyte proliferation during traumatic brain injury [21,23].

### 3.2. Astrocyte Activation and Proliferation Exhibits a Transient Time Course in the Hippocampus During TBI

We next characterized the temporal and spatial dynamics of astrocyte activation and proliferation in the hippocampus during TBI. Adult (10-week-old) C57/BL6 male mice were subjected to CCI injury or sham surgery as previously described [18]. Hippocampal tissues were collected at 4 h, 24 h, 48 h, 72 h, and 1 week after injury to assess astrocytic responses.

To determine whether reactive astrocytes also undergo proliferation, mice received a single BrdU injection (100 mg/kg, i.p.) 4 h prior to sacrifice for labeling proliferating cells. Double immunostaining for GFAP and BrdU was used to identify proliferating astrocytes (Figure 2a–f). In sham-operated mice, BrdU-positive cells were largely restricted to the subgranular zone, reflecting basal neural stem cell proliferation, and the vast majority of GFAP-positive astrocytes were BrdU-negative, indicating minimal astrocyte proliferation under basal conditions (Figure 2a). During TBI, astrocytes exhibited increased GFAP expression at 4 h and 24 h post-injury (Figure 2b,c); however, astrocyte proliferation was minimal at these early time points. In contrast, astrocyte proliferation increased markedly at later time points. Beginning at 48 h post-injury, the number of GFAP- and BrdU-positive cells rose sharply, accompanied by a significant increase in proliferating astrocytes, reaching 9.7% at 48 h (Figure 2d,i,k) and 6.2% at 72 h (Figure 2e,j,k) in total GFAP-positive astrocytes. By 1 week post-injury, the proportion of proliferating astrocytes returned to baseline levels (Figure 2f,h). Subregional analysis showed comparable levels of astrocyte proliferation in CA1, CA3, and DG, with proliferating astrocytes in the DG again predominantly localized to the molecular layer (Figure 2h). Together, these results demonstrate that TBI induces a rapid and sustained astrocyte activation in the hippocampus, with a transient wave of astrocyte proliferation peaking primarily between 48 and 72 h post-injury, particularly within the dentate gyrus.

### 3.3. TBI Activates mTORC1 Signaling in Reactive Astrocytes

**Figure 3:**
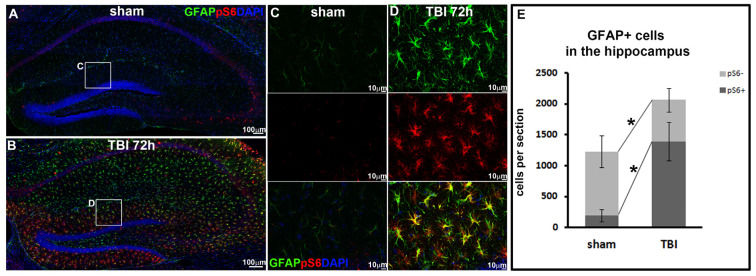
TBI activates mTORC1 signaling in reactive astrocytes. (**A**,**B**) Immunostaining with antibody against GFAP (green) and pS6 (red) demonstrated activation of mTORC1 in reactive astrocytes during TBI compared to sham (**C**,**D**) Enlarged images of white box in (**A**,**B**) respectively. (**E**) Quantification of GFAP- and pS6-positive cells in the hippocampus (*n* = 5 for each group, * *p* < 0.05).

CNS trauma largely induces astrocyte reactivation exhibited by cellular hypertrophy, gene expression alteration, and proliferation increase both around the lesion site and remote to the lesion center [24]. The beneficial and deleterious functions of reactive astrocytes are constantly debated [25,26,27,28,29,30], while the molecular mechanism mediating astrocyte reactivation remains largely unknown. Our previous study demonstrated drastic activation of mTORC1 signaling in the hippocampus during TBI, and the temporal and spatial course of mTORC1 activation strongly correlated to astrocyte reactivation at 72 h post-injury [13]. We hypothesized that mTORC1 is involved in astrocyte reactivation during TBI in the hippocampus. In the current study, to evaluate mTORC1 activation specifically in reactive astrocytes, we performed CCI on male mice to generate a moderate TBI. By immunostaining with antibodies against GFAP and pS6, we assessed mTORC1 activation specifically in reactive astrocytes in the hippocampus at the injury epicenter 72 h post-injury, the previously proved time point with a high level of astrocyte reactivation. In sham animals, we observed a low level of GFAP staining, indicating a basal level of resident astrocyte activity in the hippocampus, and their morphologies are featured as long and thin processes (Figure 3A,C). Very few of the resident astrocytes co-labeled with a low level of mTORC1 activity showed as weak pS6 staining (Figure 3A,C). At 72 h post-injury, GFAP staining showed a dramatic reactivation of astrocytes in the hippocampus, exhibiting as greatly increased GFAP intensity and hypertrophic cell morphology (Figure 3B,D). The hypertrophic reactive astrocytes presented enlarged soma and thick processes compared to resident astrocytes, and largely increased their mTORC1 activation, featured as strong pS6 co-staining (Figure 3B,D). Our quantification confirmed that in sham animals, only 1226 ± 356 resident astrocytes per section were visible by GFAP staining in the hippocampus; within them only 15.6% ± 7.3% were showing weak mTORC1 activation (Figure 3E), consistent with mTORC1 involvement in various basal cellular events [31]. At 72 h post-injury, the number of reactive astrocytes significantly increased to 2062 ± 501 per section in the hippocampus (*p* = 0.026), within them 67.2% ± 10.3% were co-labeled with strong pS6 staining demonstrating robust activation of mTORC1 signaling in reactive astrocytes (*p* = 0.003, Figure 3E). Our data indicated that activation of mTORC1 signaling is likely involved in astrocyte reactivation during TBI in the hippocampus.

### 3.4. TBI Activates mTORC1 Signaling in Proliferative Reactive Astrocytes

**Figure 4:**
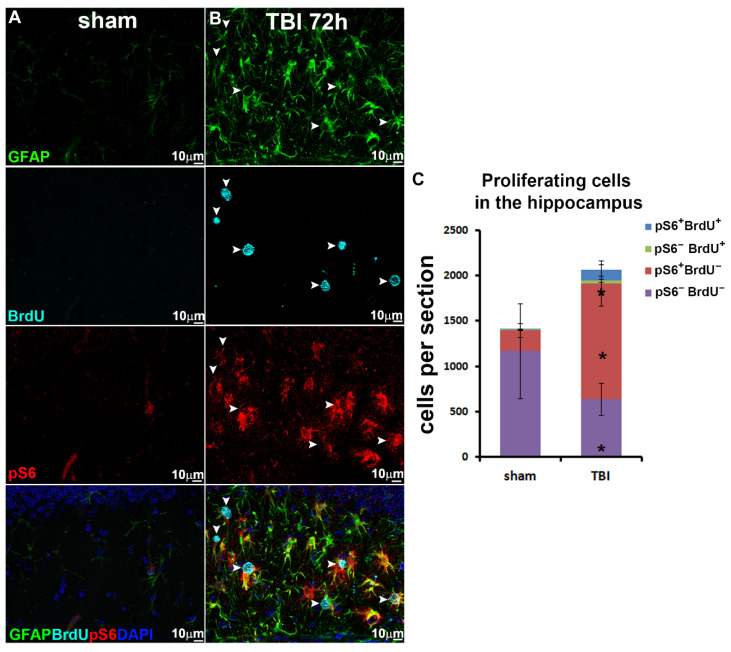
TBI activates mTOR signaling in proliferative reactive astrocytes. (**A**,**B**) Immunostaining with antibody against GFAP (green), BrdU (cyan) and pS6 (red) demonstrated activation of mTORC1 in proliferative reactive astrocytes during TBI compared to sham (white arrow head: proliferating reactive astrocyte with pS6 expression). (**C**) Quantification of BrdU- and pS6-positive cells in the hippocampus (*n* = 5 for each group, * *p* < 0.05).

Since 72 h post-injury is also the time point when reactive astrocytes show high level of proliferation, we further investigated mTORC1 activation especially in proliferative reactive astrocytes. To label reactive astrocytes proliferation, we administered BrdU to sham and injured mice at 4 h before tissue collection. By immunostaining with antibodies against GFAP, BrdU, and pS6, we evaluated mTORC1 activation selectively in proliferative reactive astrocytes. In sham animals, very limited resident astrocytes were proliferating (8 ± 1 cells per section), representing 0.58% ± 0.25% of total resident astrocytes, within which even fewer were co-labeled with pS6 staining (1 ± 1 cells per section, Figure 4A,C). At 72 h post-injury, a large amount of reactive astrocyte proliferated (145 ± 76 cells per section, *p* = 0.035), representing 6.85% ± 0.29% of total reactive astrocytes, within which a significantly larger proportion were co-stained with pS6 (114 ± 65 cells per section, *p* = 0.039, Figure 4B,C). Our data demonstrated that mTORC1 signaling was strongly activated in proliferative reactive astrocytes. Notably, a large number of mTORC1 active reactive astrocytes were not labeled by BrdU (15.79% ± 7.43% in sham animals vs. 61.79% ± 7.57% in TBI animals, *p* = 0.0017, Figure 4C), indicating potential mTORC1 involvement in other aspects of astrocyte reactivation besides proliferation as well.

### 3.5. Inhibition of mTORC1 Signaling During TBI Reduced Reactive Astrocyte Proliferation Without Affecting Hypertrophy

To determine whether mTORC1 signaling mediates astrocyte reactivation during TBI, we administered rapamycin (2 mg/kg) to injured mice to inhibit mTORC1 activity. A dose of BrdU was injected 4 h before tissue collection to label proliferating cells.

**Figure 5:**
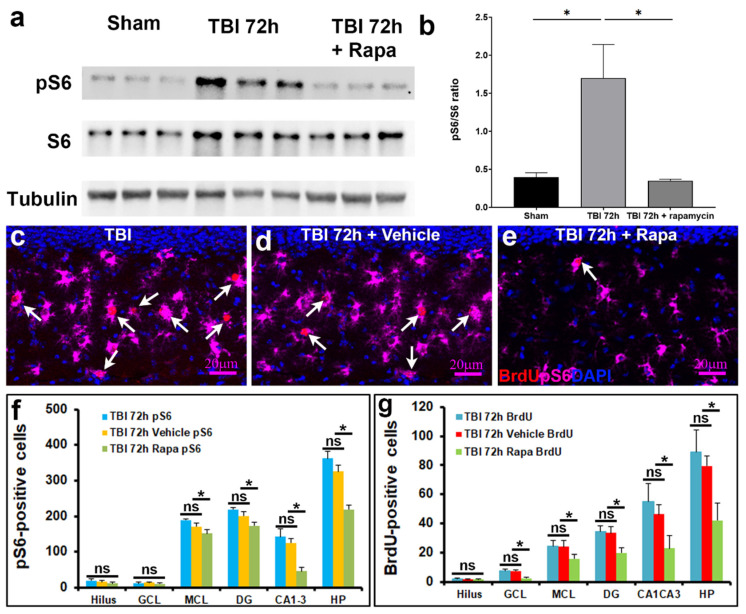
mTOR inhibition during TBI reduced reactive proliferative astrocytes. (**a**) Representative immunoblots of total S6 and phosphorylated S6 in sham, TBI-vehicle and TBI-rapamycin treated groups. (**b**) Quantification of protein expression showing reduction in pS6/S6 ratio in rapamycin-treated group when compared to vehicle-treated group during TBI (*n* = 3 for each group). (**c**–**e**) Immunostaining of BrdU (red) and pS6 (magenta) in the hippocampus of TBI-, vehicle- and rapamycin-treated groups (white arrow: co-labeling of BrdU and pS6 positive proliferating cells). (**f**) Quantification of pS6-positive cells and (**g**) BrdU-positive cells in hippocampus and its sub-regions: CA1 + CA3 and DG and its sub-regions, molecular layer (ML), granule cell layer (GCL), and hilus (*n* = 5 for each group, * *p* < 0.05). ns: not significant. The original western blot images can be found in Supplementary Materials.

Western blot analysis of hippocampal lysates revealed that rapamycin treatment markedly reduced pS6 levels in TBI mice without affecting total S6 expression, confirming effective inhibition of mTORC1 signaling in the injured hippocampus (Figure 5a,b). Consistent with these findings, immunofluorescence analysis demonstrated robust pS6 immunoreactivity in the hippocampus during TBI, which was predominantly localized to GFAP-positive astrocytes. Rapamycin treatment substantially attenuated pS6 staining in GFAP-positive cells, indicating that mTORC1 activation occurs in reactive astrocytes during injury and is sensitive to pharmacological inhibition (Figure 5c–f). Furthermore, rapamycin treatment significantly reduced the number of proliferating cells in the hippocampus compared with vehicle-treated TBI animals (Figure 5c–e,g), supporting a critical role for astrocytic mTORC1 signaling in injury-induced cell proliferation.

**Figure 6:**
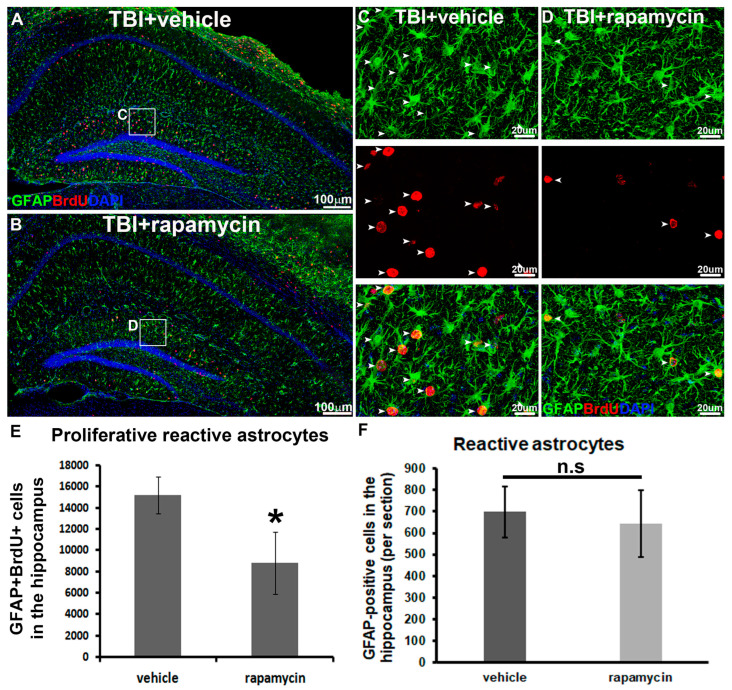
mTOR inhibition during TBI reduces astrocyte proliferation without affecting hypertrophy. (**A**,**B**) Immunostaining of GFAP (green) and (BrdU) in the hippocampus in TBI-vehicle- and rapamycin-treated groups. (**C**,**D**) Enlarged images of white box in (**A**,**B**) respectively (white arrow head: proliferating reactive astrocyte). (**E**) Quantification of proliferative reactive astrocytes showing reduction in proliferative reactive astrocytes in rapamycin-treated group when compared to the vehicle-treated group during TBI. (**F**) Quantification of GFAP-positive cells in the hippocampus showing no significant changes in reactive astrocytes in rapamycin-treated group when compared to the vehicle-treated group during TBI (*n* = 5 for each group, * *p* < 0.05). n.s: not significant.

Rapamycin treatment did not alter the overall pattern of GFAP expression in the hippocampus compared to vehicle-treated animals (Figure 6A,B), indicating that mTORC1 inhibition does not interfere with astrocyte hypertrophy or general activation. However, the proliferation of reactive astrocytes was markedly reduced, decreasing from 15,188 ± 1708 cells per hippocampus in vehicle-treated TBI animals to 8800 ± 2944 cells per hippocampus in rapamycin-treated TBI animals (*p* = 0.031, Figure 6E). These findings demonstrate that mTORC1 activity is required for the proliferation of reactive astrocytes during TBI.

Moreover, rapamycin-treated animals exhibited reactive astrocyte morphology comparable to that of vehicle-treated animals (Figure 6C,D). Quantification of GFAP-positive cells in the hippocampus revealed no significant difference between the vehicle-treated and rapamycin-treated groups (Figure 6F). These results indicate that mTORC1 inhibition selectively affects astrocyte proliferation without altering hypertrophy. Together, the data demonstrate that mTORC1 plays a primary role in mediating reactive astrocyte proliferation during TBI.

## 4. Discussion

Traumatic brain injury triggers a rapid and multifaceted response from astrocytes, particularly within the hippocampus, a brain region essential for learning and memory [32]. In this study, we provide a detailed temporal and spatial analysis of astrocyte reactivity during controlled cortical impact and identify mTORC1 signaling as a key intracellular pathway associated with these changes. Together, our findings reveal a coordinated sequence of astrocytic responses—early hypertrophy, a brief period of proliferation, and robust mTORC1 activation—that deepens our understanding of how astrocytes contribute to the pathophysiology of TBI.

Astrocyte activation occurred dramatically early during injury. In sham animals, astrocytes displayed the expected resting morphology, characterized by sparse GFAP expression and thin, delicate processes consistent with a homeostatic surveillance state. In contrast, injured animals showed a pronounced increase in GFAP-positive astrocytes as early as four hours post-injury. These cells exhibited all classic features of reactive astrocytes such as enlarged cell bodies, thickened processes, and intense GFAP immunoreactivity [33,34]. This rapid transformation reinforces the notion that astrocytes are among the first responders to CNS trauma.

Importantly, astrocyte reactivity followed a clear biphasic pattern. GFAP-positive cell numbers rose sharply within hours of injury, remained elevated through 48 h and 72 h before gradually returning to baseline levels by one week and later. This temporal trajectory closely mirrors prior reports of astrocytic responses to TBI and highlights the dynamic nature of astrocyte reactivation rather than a static or uniform process. The spatial heterogeneity of astrocyte responses within the hippocampus further highlights the complexity of astrocyte reactivity. Among hippocampal sub-regions, the dentate gyrus, particularly its molecular layer, emerged as the most responsive compartment. Astrocyte activation in CA1 and CA3 regions occurred rapidly but resolved relatively quickly, whereas the dentate gyrus exhibited both early onset and prolonged reactivity. Given the known vulnerability of dentate gyrus to metabolic stress and excitotoxic injury, this prolonged astrocyte response may be especially relevant to the cognitive deficits commonly observed during TBI [35].

Although hypertrophy was the earliest and most persistent feature of astrocyte activation, proliferation followed a much more restricted timeline. Under normal conditions, hippocampal astrocytes rarely divide, as reflected by minimal BrdU incorporation in sham animals. Early during injury, despite robust increases in GFAP expression, astrocyte proliferation remained at baseline levels, indicating that structural and molecular activation precedes cell cycle entry. This temporal separation underscores the stepwise nature of astrocyte reactivity. However, a marked shift occurred at 48 and 72 h post-injury, when astrocyte proliferation increased nearly tenfold. This proliferative burst was evident across hippocampal regions but was most pronounced in the dentate gyrus, particularly within the molecular layer. In contrast, the granule cell layer and hilus exhibited more modest increases. By one week post-injury, astrocyte proliferation had largely subsided, even though GFAP expression remained elevated. These observations suggest that astrocyte proliferation is both robust and transient, confined to a narrow window during the early secondary injury phase.

The functional role of this brief proliferative response remains an open question. In spinal cord injury, proliferating astrocytes contribute to glial scar formation, which can serve both protective and inhibitory roles [29,36]. However, in regions distant from the primary lesion, such as the hippocampus, classic scar formation is neither typical nor required. Instead, proliferating astrocytes in the hippocampus may support alternative functions, including modulation of inflammation, stabilization of synaptic networks, metabolic support, or remodeling associated with neurogenesis. Our current findings provide a background to dissect these possibilities through selective targeting of proliferative astrocyte populations.

A key finding of our study is the robust activation of mTORC1 signaling in reactive astrocytes during TBI. At 72 h post-injury, a time point marked by both hypertrophy and peak proliferation, the majority of astrocytes exhibited strong pS6 immunoreactivity, which is a canonical indicator of mTORC1 activity. In contrast, astrocytes in sham animals showed minimal pS6 labeling, consistent with low basal mTORC1 activity under physiological conditions. These findings are consistent with prior work demonstrating that mTOR signaling is activated in a time- and region-dependent manner during TBI, including delayed activation of S6 in reactive astrocytes within the hippocampus between 24 and 72 h post-injury [37].

Notably, mTORC1 activation was particularly enriched in proliferating astrocytes. A large proportion of BrdU-positive astrocytes also expressed pS6, indicating a close association between mTORC1 signaling and astrocyte cell cycle entry. This relationship is consistent with the well-established role of mTORC1 in regulating cellular growth, metabolism, and proliferation. The tight temporal alignment between maximal mTORC1 activation and peak astrocyte proliferation strongly supports a model in which mTORC1 signaling enables the metabolic and biosynthetic demands required for astrocyte division during injury. Littlejohn et al. showed that pharmacological inhibition of mTOR with rapamycin can modulate post-traumatic cellular responses, including altering BrdU-positive cell population, and influence astrocyte-associated processes in the hippocampus [37].

At the same time, not all astrocytes with elevated mTORC1 activity were proliferating. A substantial subset of pS6-positive astrocytes lacked BrdU incorporation, suggesting that mTORC1 also supports non-mitotic aspects of astrocyte reactivity. Previous studies have implicated mTORC1 in cytoskeletal remodeling [38], hypertrophic growth [39], translational control [40], and metabolic adaptation [41]. These processes align well with the morphological and molecular changes observed in reactive astrocytes during TBI. Thus, mTORC1 appears to function as a versatile regulator that coordinates both proliferative and non-proliferative components of astrocyte activation.

Additionally, we pharmacologically inhibited mTORC1 with rapamycin to obtain further mechanistic insight. Rapamycin effectively suppressed pS6 signaling in GFAP-positive astrocytes and significantly reduced reactive astrocyte proliferation in the hippocampus during TBI, without altering overall GFAP expression levels or hypertrophic morphology. These findings are in line with previous reports showing mTOR inhibition attenuates astrocyte proliferation and reactive gliosis, and that rapamycin can modulate astrogliosis and neuroinflammatory responses during TBI [42]. These results indicate that mTORC1 activity is required for astrocyte cell-cycle re-entry but not required for the initial hypertrophic activation of astrocytes. This dissociation supports the concept that astrocyte reactivity comprises multiple mechanistically distinct components, hypertrophy and proliferation, which are differentially regulated at the molecular level. These results are in agreement with previous studies showing that mTOR inhibition restrains astrocyte proliferation and migration without fully abolishing reactive gliosis [43].

Together, these findings position mTORC1 as a central regulator of reactive astrocyte proliferation in the hippocampus during TBI. Given the dual protective and potentially maladaptive roles of reactive astrocytes, selective modulation of mTORC1 signaling may represent a therapeutic strategy to fine-tune astrocyte responses during brain injury. Future studies will be required to determine how astrocyte-specific mTORC1 signaling influences hippocampal neurogenesis, synaptic plasticity, and long-term cognitive outcomes during TBI, as well as to define the upstream signals that trigger mTORC1 activation in astrocytes during injury.

## 5. Conclusions

In summary, our study defines a clear temporal and mechanistic framework for astrocyte reactivity in the hippocampus during TBI. We show that astrocytes respond rapidly to injury with pronounced hypertrophy, followed by a short and well-defined wave of proliferation that peaks during the acute post-injury phase and then resolves. This proliferative response is not a generic feature of astrocyte activation but instead represents a distinct and tightly regulated component of the reactive program. A central finding of our work is that mTORC1 signaling is robustly activated in hippocampal astrocytes during TBI and is specifically required for their proliferative response. Pharmacological inhibition of mTORC1 effectively suppressed astrocyte proliferation without altering hypertrophy or overall GFAP expression, demonstrating that these two hallmarks of astrogliosis are molecularly separable. These results position mTORC1 as a key regulator of astrocyte cell-cycle re-entry during injury, rather than a general driver of astrocyte activation. By distinguishing hypertrophic activation from proliferation and identifying mTORC1 as a critical signaling node controlling the latter, our study advances our understanding of astrocyte biology in the injured hippocampus. Given the central role of the hippocampus in cognition and the lasting neurological consequences of TBI, selectively targeting astrocyte proliferation through mTORC1 modulation may offer a promising strategy to shape post-traumatic glial responses without broadly suppressing astrocyte functions that are essential for tissue repair and homeostasis. Future work will be necessary to determine how this transient proliferative astrocyte population influences hippocampal circuit remodeling, neurogenesis, and long-term functional recovery during traumatic brain injury.

## Data Availability

The original contributions presented in this study are included in the article/Supplementary Material. Further inquiries can be directed to the corresponding author.

## Supplementary Materials

The following supporting information can be downloaded at: https://www.mdpi.com/article/10.3390/biom16040555/s1, Supplementary Material: The original western blot images.
