# Supplementary figures and images for: mTOR Activation Is Required for the Proliferation of Reactive Astrocytes in the Hippocampus During Traumatic Brain Injury
Source: Biomolecules. 2026 Apr 9;16(4):555. doi: 10.3390/biom16040555 (PMC13113957; doi:10.3390/biom16040555)

The original blot images

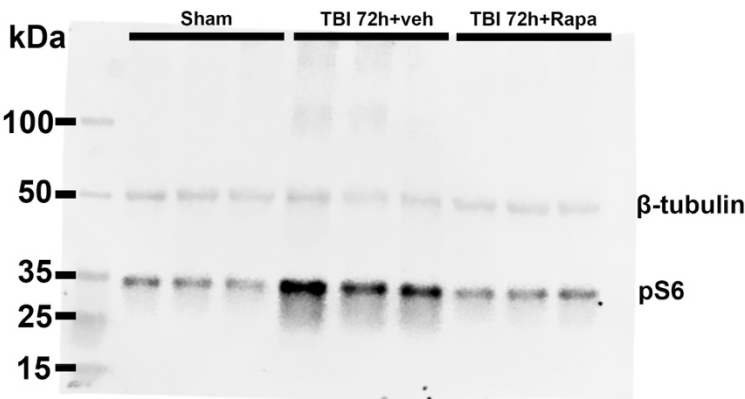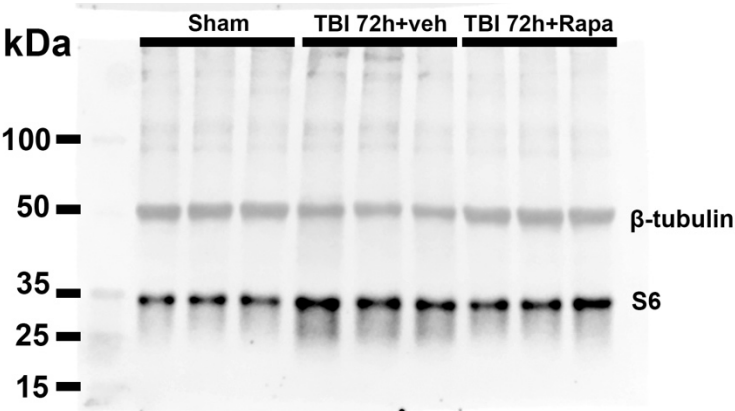

Supplement: Supplementary file 1 [file biomolecules-16-00555-s001.zip › biomolecules-4168879-supplementary.pdf]
